# *Factor* 8 Gene Mutation Spectrum of 270 Patients with Hemophilia A: Identification of 36 Novel Mutations

**DOI:** 10.4274/tjh.galenos.2020.2019.0262

**Published:** 2020-08-28

**Authors:** Tahir Atik, Esra Işık, Hüseyin Onay, Bilçağ Akgün, Moharram Shamsali, Kaan Kavaklo, Melike Evim, Gülen Tüysüz, Namık Yaşar Özbek, Fahri Şahin, Zafer Salcıoğlu, Canan Albayrak, Yeşim Oymak, Ekrem Ünal, Fatma Burcu Belen, Ebru Yılmaz Keskin, Can Balkan, Birol Baytan, Alphan Küpesiz, Vildan Culha, Tuba Nur Tahtakesen Güçer, Adalet Meral Güneş, Ferda Özkınay

**Affiliations:** 1 Ege University Faculty of Medicine, Department of Pediatrics, Division of Pediatric Genetics, İzmir, Turkey; 2 Ege University Faculty of Medicine, Department of Medical Genetics, İzmir, Turkey; 3 Ege University Institute of Health Sciences, Division of Health Bioinformatics, İzmir, Turkey; 4 Ege University Faculty of Medicine, Department of Pediatrics, Division of Pediatric Hematology, İzmir, Turkey; 5 Uludağ University Faculty of Medicine, Department of Pediatrics, Division of Pediatric Hematology, Bursa, Turkey; 6 Akdeniz University Faculty of Medicine, Department of Pediatrics, Division of Pediatric Hematology, Antalya, Turkey; 7 University of Health Sciences Turkey Ankara Pediatric Hematology Oncology Training and Research Hospital, Clinic of Pediatric Hematology, Ankara, Turkey; 8 Ege University Faculty of Medicine, Department of Internal Medicine, Division of Hematology, İzmir, Turkey; 9 İstanbul Kanuni Sultan Süleyman Training and Research Hospital, Clinic of Pediatric Hematology and Oncology, İstanbul, Turkey; 10 Ondokuz Mayıs University Faculty of Medicine, Department of Pediatric Hematology and Oncology, Samsun, Turkey; 11 Dr. Behcet Uz Children’s Hospital, Division of Pediatric Hematology, İzmir, Turkey; 12 Erciyes University Faculty of Medicine, Department of Pediatrics, Division of Pediatric Hematology, Kayseri, Turkey; 13 Katip Çelebi University Faculty of Medicine, Department of Pediatrics, Division of Pediatric Hematology, İzmir, Turkey; 14 Süleyman Demirel University Faculty of Medicine, Department of Pediatrics, Division of Pediatric Hematology, Isparta, Turkey; # Equal contributors

**Keywords:** Hemophilia A, F8 gene, Mutation, Inhibitors, Intron 22 inversion, Turkey

## Abstract

**Objective:**

Hemophilia A (HA) is the most severe X-linked inherited bleeding disorder caused by hemizygous mutations in the *factor 8 (F8)* gene. The aim of this study is to determine the mutation spectrum of the *F8* gene in a large HA cohort from Turkey, and then to establish a phenotype-genotype correlation.

**Materials and Methods:**

All HA cases (270 patients) analyzed molecularly in the Ege University Pediatric Genetics Molecular Laboratory between March 2017 and March 2018 were included in this study. To identify intron 22 inversion (Inv22), intron 1 inversion (Inv1), small deletion/insertions, and point mutations, molecular analyses of *F8* were performed using a sequential application of molecular techniques.

**Results:**

The mutation detection success rate was 95.2%. Positive Inv22 was found in 106 patients (39.3%), Inv1 was found in 4 patients (1.5%), and 106 different disease-causing sequence variants were identified in 137 patients (50.6%). In 10 patients (3.7%), amplification failures involving one or more exonic regions, considered to be large intragenic deletions, were identified. Of 106 different *F8* mutations, 36 were novel. The relationship between *F8* genotype and inhibitor development was considered significant.

**Conclusion:**

A high mutation detection rate was achieved via the broad molecular techniques applied in this study, including 36 novel mutations. With regard to mutation types, mutation distribution and their impact on clinical severity and inhibitor development were found to be similar to those previously reported in other hemophilia population studies.

## Abstract (tr)

**Amaç:**

Hemofili A (HA), *faktör 8 (F8)* genindeki hemizigot mutasyonların neden olduğu X’e bağlı kalıtsal kanama bozukluğudur. Bu çalışmanın amacı, Türkiye’den büyük bir HA kohortunda F8 geninin mutasyon spektrumunu belirlemek ve fenotip-genotip korelasyonu oluşturmaktır.

**Gereç ve Yöntemler:**

Mart 2017-Mart 2018 tarihleri arasında Ege Üniversitesi Pediatrik Genetik Moleküler Laboratuvarı’nda moleküler olarak analiz edilen tüm HA hastaları (270 hasta) çalışmaya dahil edildi. “İntron 22 inversiyonu” (Inv22), “intron 1 inversiyonu” (Inv1), “küçük delesyon/duplikasyonlar” ve “nokta mutasyonları” tanımlamak için *F8*’in moleküler analizleri, uygun bir algoritma kullanılarak gerçekleştirildi.

**Bulgular:**

Mutasyon saptama başarı oranı %95,2’ydi. Yüz altı hastada (%39,3) Inv22 pozitif, 4 hastada (%1,5) Inv1, 137 hastada (%50,6) Yüz altı farklı hastalık yapıcı sekans varyantı saptandı. On hastada (%3,7), büyük intragenik delesyonlar olduğu öngörülen bir veya daha fazla ekzonik bölgeyi içeren amplifikasyon başarısızlığı tespit edildi. 106 farklı F8 mutasyonundan 36’sı ilk kez bu çalışmada saptandı. F8 genotipi ile inhibitör gelişimi arasındaki ilişki anlamlı kabul edildi.

**Sonuç:**

Bu çalışmada gerçekleştirilen moleküler teknikler ile yüksek mutasyon tespit oranı elde edilmiştir; 36 yeni mutasyon saptanmıştır. Mutasyon tipleri ile ilgili olarak, mutasyon dağılımı ve bunların klinik şiddeti ve inhibitör gelişimi üzerindeki etkisi, farklı hemofili popülasyon çalışmalarında daha önce bildirilenlere benzer bulunmuştur.

## Introduction

Hemophilia A (HA) is an X-linked disease with a prevalence of approximately 1 in 5000 males, and it is the most severe inherited bleeding disorder. The clinical phenotype of HA is classified as severe (FVIII:C <1%), moderate (FVIII:C 1%-5%), and mild (FVIII:C >5%) in accordance with the level of coagulant activity of FVIII (FVIII:C) [1].

The coagulation *factor 8* gene (*F8*) is one of the largest genes in the genome, spanning 186 kb, consisting of 26 exons, and being localized at Xq28 [2,3]. More than 3000 unique mutations have been recorded across both the HA Mutation Database (HAMSTeRS) and the Human Gene Mutation Database (HGMD) [4,5,6]. Mutations are classified into three groups: large rearrangements (intron 22 inversion, intron 1 inversion), intragenic deletions or insertions, and single-nucleotide variants (missense, nonsense, and splice site). In severe HA the most common gene defect is an intron 22 inversion, which is responsible for 40%-50% of cases. However, taking all phenotypes into consideration, point mutations are found in about 47%, making them the most prolific. The other* F8* gene variants, such as intron 1 inversion (Inv) and large deletions, are seen less frequently [7,8,9].

The* F8* genotype is reported to be associated with clinical severity, risk of inhibitor formation, and response to immune tolerance therapy. Taking this into account, mutation analysis of the *F8* gene is crucial for prediction of disease severity, choice of appropriate treatment, and optimal genetic counseling [10]. The aim of this study is to determine the mutation spectrum of the *F8* gene in HA patients and then to establish a phenotype-genotype correlation.

## Materials and Methods

### Participants

All HA cases (270 patients) analyzed molecularly in the Ege University Pediatric Genetics Molecular Laboratory between March 2017 and March 2018 were included in this study. Demographic features, factor VIII:C levels, and inhibitor status were all obtained from medical records retrospectively. The clinical severity of the patients was classified into three groups (severe, moderate, and mild) in accordance with their factor VIII:C levels, which had been measured previously using a standard one-stage clotting assay. The FVIII inhibitor titers of all HA patients in this study were quantified using the Nijmegen modification of the Bethesda assay [11]. Informed consent for all molecular studies was obtained from either the patient directly or from their guardians. The study was approved by the Erciyes University Ethics Review Committee.

### Molecular Genetic Analysis

Genomic DNA was extracted from 2 mL of peripheral blood in EDTA using a Gentra Puregene Blood Kit (QIAGEN), in accordance with the manufacturer’s instructions. Using the inverse-shifting polymerase chain reaction (PCR) method [12], all patients were first screened for intron 22 inversion (Inv22) with the HA Genotyping Kit, Part A (MultiGen Healthcare). Any negative results were then tested for Inv1 via multiplex PCR. In patients found to be negative for both Inv22 and Inv1, a sequencing analysis of all the coding regions and exon-intron boundaries of the *F8* gene was then performed. The sequence analysis was performed on an Illumina MiSeq or MiniSeq platform using the HA Genotyping Kit, Part B (MultiGen Healthcare).

### Variant Analysis

Sequence variants were interpreted in accordance with the American College of Medical Genetics and Genomics guidelines [13]. All identified *F8* gene variants with a frequency of less than 1% in public databases were selected. Databases included NCBI dbSNP build141 (http://www.ncbi.nlm.nih.gov/SNP/), the 1000 Genomes Project (http://www.1000genomes.org/), the Exome Aggregation Consortium (ExAC) (http://exac.broadinstitute.org/), and the NHLBI Exome Sequencing Project (ESP) Exome Variant Server (http://evs.gs.washington.edu/EVS/). Selected variants were then checked against the HAMSTeRS database (http://www. HAMSTeRS.ac.uk/) and the HGMD [5,6]. The impact of novel variants on the protein structure was then classified using several in silico prediction tools such as MutationTaster, Polyphen-2, and SIFT [14,15,16]. Conservation of residues across species was evaluated using the PhyloP algorithm and GERP [17,18].

The mutations found in this study were classified as either high-risk (Inv22, Inv1, large deletions, point mutations including nonsense, frameshifts) or low-risk *F8* genotypes (missense variants, inframe deletion/insertions and splice mutations). As outlined in the RODIN study, which suggested an association between the *F8* genotype and its impact on inhibitor development, the mutations identified in this study were also classified within the same parameters [10,19].

### Statistical Analysis

IBM SPSS Statistics for Windows 21.0 (IBM Corp., Armonk, NY, USA) was used for all analyses. Comparisons were made using the Fisher exact test and p<0.05 was considered significant.

## Results

### Mutation Spectrum and Novel Mutations

Molecular analysis was performed for 270 HA patients (age at diagnosis: 7.9±5.27 months) from unrelated families. Of the patients, 269 were boys. In 106 HA patients (39.3%), Inv22 mutation was found. Following the second step, Inv1 was found in 4 HA patients (1.5%). The remaining Inv22- and Inv1-negative patients were then analyzed for *F8* sequence variations and a disease-causing variant was found in 137 (50.6%). In patients with no *F8* gene mutation, sequence views were reevaluated using IGV [20]. In 10 patients (3.7%), amplification failures involving one or more exonic regions, considered as large intragenic deletions, were identified. The sequence analysis of the patients in whom these deletions were found was then resequenced, and the same results were confirmed (del ex1, del ex2-9, del ex7-13 (in two unrelated families), del ex9, del ex11-12, del ex12, del ex14, del ex15-22, del ex26). After all molecular analysis steps, no mutations were found in 13 HA patients (4.8%). Across the whole study group, considering Inv22, Inv1, and *F8* sequence analysis, the mutation detection success rate was 95.2%.

A total of 106 different likely pathogenic and pathogenic variants were identified within 137 families. Of the variants, 56 (52.9%) were missense, 18 (16.9%) nonsense, 25 (23.7%) frameshift, 6 (5.6%) splice site, and 1 (0.9%) inframe deletion. Among the 106 mutations, 36 mutations in 42 families were novel (33.9%). Of the 36 novel mutations, 16 (44.5%) were frameshift, 15 (41.7%) missense, and 5 (13.8%) nonsense. The novel mutations identified in this study, including their distribution in exonic and domain levels, are given in Table 1. A list of all mutations detected in this study is given as supplemental data in the Appendix.

**Figure 1:**
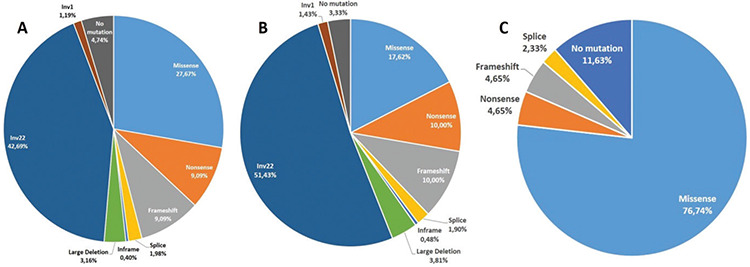
Frequencies of different types of *F8* DNA variants detected in all HA (A), severe HA (B), and mild/moderate HA (C) cases. HA: Hemophilia A

Taking the clinical severity of the 270 HA patients into consideration, 221 (81.9%) cases were severe, with 49 (18.1%) moderate or mild. The mutation spectra of both severe and/or mild/moderate HA groups are given in Figure 1.

One of the patients in the study group was an 11-month-old girl, admitted to the hospital due to a right occipital fracture and epidural hematoma [21]. She was born to consanguineous parents, and her father had severe HA. Her coagulation test results were found compatible with severe HA. In this patient, the homozygous variant c.608T>C (L203P) was found. Segregation analysis showed that the father had the same mutation hemizygously and the mother heterozygously.

Of all HA patients with a causative mutation identified in this study, 67.3% (173 of 257) were classified as high-risk. Among severe HA patients, this frequency increased to 78.9% (168 of 213).

The association between mutation risk group and clinical severity was found to be statistically significant (p<0.001).

### Inhibitor Development

Inhibitor status of all but 2 HA patients (1 with severe and 1 with mild/moderate HA) was evaluated in this study. The frequency of inhibitor-positive patients was found to be 14.1% (38 of 268). When only severe HA patients were taken into consideration, this frequency was 16.7% (37 of 220). From among the mild/moderate HA group, only one patient was found to be inhibitor-positive (2%; 1 of 48).

In the patients with Inv22, the frequency of inhibitor positivity was 23.6% (25 of 106), significantly higher than in those without Inv22 (p=0.001). Two of 4 patients (50%) with Inv1 showed inhibitor positivity.

In the high-risk mutation group, inhibitor positivity was found in 35 of 173 patients (20.2%). In the low-risk mutation group, inhibitor development was detected in only 3 of 84 patients (3.6%). The association between mutation risk group and inhibitor development was statistically significant (p<0.001).

## Discussion

Since the discovery of the *F8* gene in 1984, a number of studies evaluating the *F8* gene mutation spectrum of patients have been published. From 846 families with severe and nonsevere hemophilia, Oldenburg et al. [22] showed intron 22 inversion to be responsible for 35.7% of cases, point mutations for 47.5%, and small deletion/insertions for 10.2%. Intron 1 inversion, large deletions, and splice site mutations were rarely found. In our study, the mutation detection rate in the *F8* gene was 95.2%. This was achieved through following a protocol that involved up to three procedures: Inv22, Inv1, and *F8* sequencing analyses. The frequencies of mutation types in our study are similar to those found in previous studies [7,23,24,25].

In mild/moderate HA cases, missense mutations are the major mutation type with a frequency of 70%-80% [4]. In this study, we found the frequency of missense mutations to be highest (77%) among mild/moderate HA patients, supporting earlier studies.

Studies from Turkey evaluating the *F8* mutation spectrum in HA patients are limited. In 1999, El-Maarri et al. [26] investigated intron 22 inversions using Southern blot analysis in 141 HA patients from Turkey. Intron 22 inversion mutation was found in 29% of all HA cases and in 42% of the severe HA cases. In another study, the mutation detection rate of DNA sequencing in intron 22 inversion-negative patients was reported as 61%, with 36 different *F8* gene mutations being detected [27]. In these previous studies from Turkey, patient numbers were limited and a complete molecular diagnosis algorithm was not followed. In terms of patient numbers, this is the largest study from Turkey and it also includes the broadest range of molecular testing techniques.

Large deletions are responsible for 3% of severe HA cases. To date, about 265 different large deletions (>50 bp) in the *F8* gene have been recorded in the HAMSTeRS database [5]. Multiplex ligation-dependent probe amplification (MLPA) is a standard test used for the detection of large deletions in the *F8* gene. MLPA analysis was not available in our laboratory; however, following the reanalysis of the *F8* gene due to amplification failures in 10 patients, large deletions were considered. In X-linked diseases it has been shown that, in males, amplification failure in PCR may indicate deletion mutations. However, MLPA analysis should be performed to confirm the hemizygous deletions. Furthermore, due to the existence of the other X chromosome with wild type, *F8* gene MLPA analysis is also necessary for detecting female deletion carriers.

Currently, more than 3000 unique mutations have been recorded in the databases of HAMSTeRS and HGMD [5,6]. In this study, we found 36 different novel mutations in 42 unrelated families. Interestingly, a novel specific missense mutation (c.608T>C) in 5 unrelated families has been reported here for the first time, suggesting a founder effect. We reinvestigated the family history from these patients to evaluate them for a common ancestral region. We identified no consanguinity between these families. Three of them were from the Aegean Region of Turkey and 2 from the Middle Anatolian Region. However, each family was from a different city.

Approximately 25%-30% of patients with severe HA develop inhibitors within 14 exposure days. Inhibitor risk is lower in patients with mild and moderate disease than those with severe disease. However, these patients still developed inhibitors at an incidence reported as 6.7% by the 50^th^ exposure day [10]. In our study of mild/moderate HA patients, the risk of inhibitor development was also significantly low (2%) when compared to the risk of severe HA. It has been reported that there is a firm correlation between genotype and inhibitor development in HA patients. The incidence of inhibitors is greatest in patients with disruptive structural variations such as large multi-exon deletions (67%-88%), while being comparatively lower in those with HA due to missense variants (<12%) [10]. Data from the recent SIPPET (Survey of Inhibitors in Plasma-Product Exposed Toddlers) study showed the risk of inhibitor formation to be highest in those with variants predicted as being null. No correlation between inhibitor risk and type of product used was observed [28]. These results support HA genotype being a strong driver of inhibitor formation, with variants resulting in little or no protein synthesis putting patients most at risk. Consistent with these data, inhibitor formation was identified as 23.6% in patients with Inv22, 30% in those with large intragenic deletions, and 50% in those with Inv1. However, in patients with missense mutations, this rate was found to be 2.6%. By dividing the mutations into two groups, high-risk and low-risk, we were able to show the risk of inhibitor development being significantly higher in patients with high-risk mutations.

In this study, no causative *F8* gene defect could be found in 4.8% of the patients. While MLPA for large deletions/duplications could not be performed in the mutation-negative patients, amplification failures in one or more exonic regions in 10 HA patients were found, indicative of large intragenic deletions. Despite large duplications being rare molecular defects causing HA, we considered that the mutation detection rate would increase slightly following an evaluation of mutation-negative patients using MLPA. However, this is by no means a guarantee of 100% mutation identification. Several factors can lead to this situation. First, some complex gene rearrangements and intronic mutations cannot be detected using the standard molecular tests currently available. Second, type 2 von Willebrand disease and the combined deficiency of FVIII and FV can also decrease the FVIII activity level, leading to a misdiagnosis of HA. These alternatives should be considered in the differential diagnosis of cases being *F8* gene mutation-negative but having low levels of FVIII activity. Although the source of factor concentration (plasma or recombinant) is considered to be an important factor of inhibitor development in patients with HA, the type of factor concentrate was not reviewed and is considered outside of the scope of this study.

### Study Limitations

In this study, we present a large cohort of Turkish HA patients and their mutation spectrum. Our molecular analysis pipeline included intron 22 and 1 inversion analysis and DNA sequencing of all exonic regions of the *F8* gene. MLPA is a standard test used for the detection of large deletions in the *F8* gene, but unfortunately we could not perform MLPA as a part of our protocol. As an X-linked disorder, in HA, hemizygous deletions in one or more exonic regions of the *F8* gene can be determined by evaluating amplification failure in PCR and showing it in next-generation sequencing (NGS). However, NGS analysis needs to be standardized for confirmation and carrier detection.

## Conclusion

A high mutation detection rate has been achieved via the broad molecular techniques performed in this study, including 36 novel mutations. Regarding mutation types, mutation distribution, and their impact on clinical severity and inhibitor development, results were found to be similar to those reported in previous studies conducted in different hemophilia populations.

## Figures and Tables

**Appendix:**
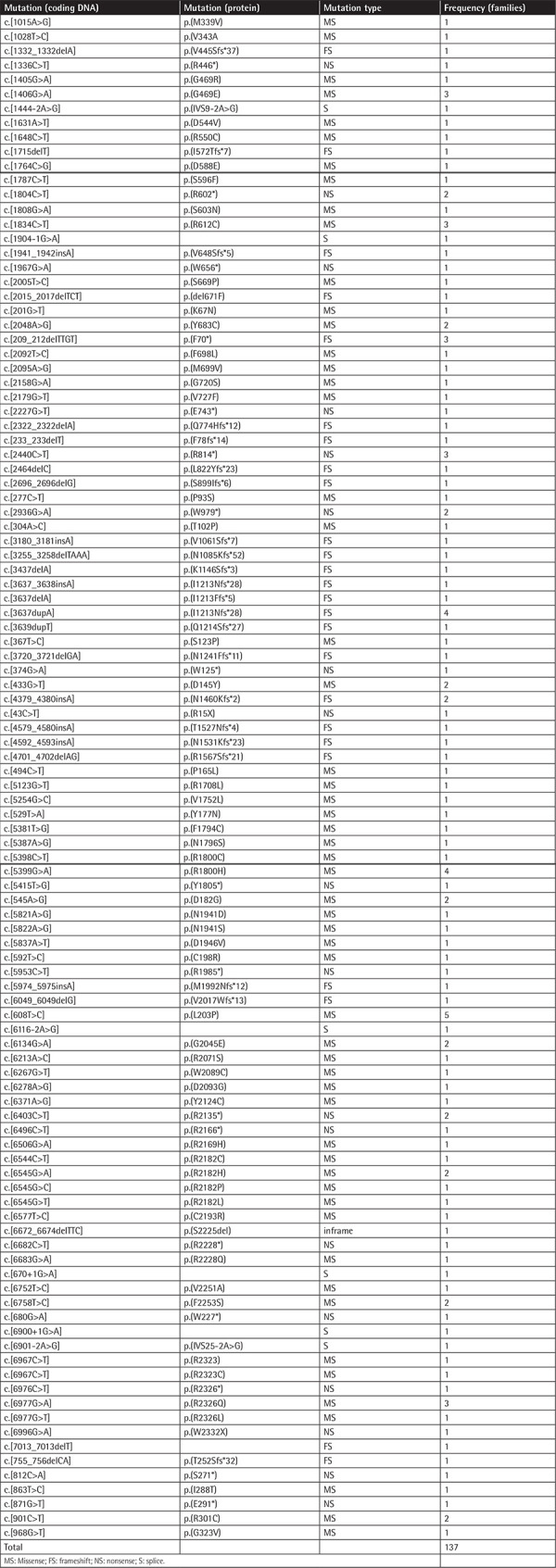
All mutations detected in this study.

**Table 1:**
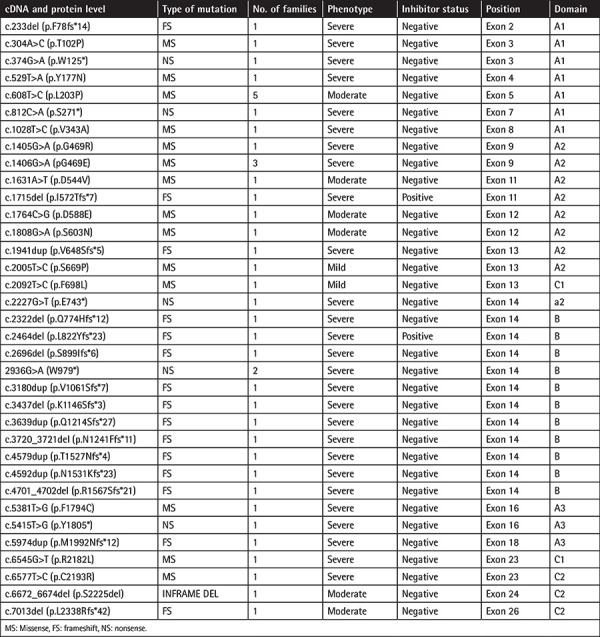
Detailed description of novel mutations detected in our patients.
